# Exosomal Long Non-coding RNAs Serve as Biomarkers for Liver Disease

**DOI:** 10.5152/tjg.2023.22741

**Published:** 2023-07-01

**Authors:** Zixuan Yang, Naping Tang, Minhui Zheng, Yan Chang

**Affiliations:** 1 China State Institute of Pharmaceutical Industry, Shanghai, China; 2 Shanghai Innostar Bio-tech Company Limited, Shanghai, China

**Keywords:** Biomarker, exosome, IncRNA, liver disease

## Abstract

Exosomes are tiny vesicles secreted by cells, with a diameter of 40-160 nm, which contain proteins, DNA, mRNA, long noncoding RNA, etc. Because of the low sensitivity and specificity of the conventional biomarkers for liver diseases, it is of utmost importance to discover novel, sensitive, specific, and non-invasive biomarkers. Exosomal long noncoding RNAs have been considered as potential diagnostic, prognostic, or predictive biomarkers in a wide range of liver pathologies. In this review, we discuss the recent progress on exosomal long noncoding RNAs that serve as potential diagnostic, prognostic, or predictive markers and molecular targets in patients with hepatocellular carcinoma, cholestatic liver injury, viral hepatitis, and alcohol-related liver diseases.

**Main Points**

- This study summarizes the exosomal long noncoding RNAs (lncRNAs) that can be used as biomarkers.
- It Introduces the mechanism of lncRNAs.
- This study compared existing biomarkers with new biomarkers and elaborated different kinds of liver diseases.

## Introduction

Extracellular vesicles (EVs) include exosomes, microvesicles, and apoptotic bodies. The production of exosomes involves the formation of intraluminal vesicles (ILVs)-containing multivesicular bodies (MVBs) and double invagination of plasma membrane (PM). intraluminal vesicles are finally secreted as exosomes (diameter = 40-160 nm) when MVBs fuse with PM and exocytosis.^1,2^ The density of an exosome is 1.15-1.19 g/ml.^2^ The tetraspanin proteins CD9, CD63, and CD81 expressed on the surface of exosomes are frequently used as the surface markers for exosomes.^3^

Most cells can secrete exosomes, which can be identified in various biofluids such as blood, saliva, semen, milk, cerebrospinal fluid, and urine.^4^ The high amount of exosomes in bodily fluids implicates their potential as a non-invasive diagnostic approach for liver diseases. Exosomes shuttle a variety of molecules, such as proteins, DNA, mRNA, miRNA, and lncRNA from parental cells to other cells.^2,5,6^ The composition of exosomes is similar to that of parental cells, and exosomes provide circulation-traceable specificity.^7^ Dynamic light scattering, transmission electron microscopy, scanning electron microscopy, and nanoparticle tracking analysis are commonly employed to assess the physical features of exosomes, including morphology, concentrations, vesicle size, and distribution.^5^

Non-invasive liquid biopsy has become a promising modality for the early diagnosis of various diseases, especially for cancer. It uses biomarkers in a sample of bodily fluids to verify the pathophysiological status of a subject. This method is widely applied in disease diagnosis, prediction, and prognosis, as well as treatment selection.^8^

Long noncoding RNA (lncRNA) is a kind of noncoding RNA (>200 nucleotides in length), which lacks a complete open reading frame, does not have the ability to encode proteins, or has a limited coding function. It has different secondary structures and can specifically bind to various nucleic acids and proteins according to the principle of complementary base pairing.^2^ Long noncoding RNA plays critical roles in gene regulation, including mRNA splicing, transcription, X-chromosome inactivation, epigenetic regulation, genomic imprinting, and nuclear-cytoplasmic trafficking.^9^

Under pathological conditions, numerous studies have reported that many exosomal lncRNAs are differentially expressed compared to normal control samples, indicating that exosomes can selectively package, secrete, and transport lncRNAs and exert specific biological functions.^10-17^ Exosomal lncRNAs are protected from RNAases; hence, they can exist stably in biofluid samples.

In this review, we focus on exosomal lncRNAs that can serve as promising biomarkers for liver diseases.

## Hepatocellular Carcinoma

Hepatocellular carcinoma (HCC) is a primary liver cancer, which accounts for a high mortality rate. It is one of the most frequently diagnosed cancer worldwide, particularly in Africa, southern Europe, and Asia. According to the statistics of the World Health Organization in 2020, HCC ranked third in mortality and sixth in incidence, causing more than 905 000 new cases and 830 000 new deaths in 2020.^18^ The occurrence of HCC is a complex process involving many risk factors, including aflatoxin B1 (AFB1) exposure, hepatitis B virus (HBV) infection or hepatitis C virus (HCV) infection, smoking, obesity, and diabetes.

Alpha-fetoprotein (AFP) remains the most commonly used biomarker for screening and diagnosing HCC. However, AFP is not secreted in all HCC cells and it is also increased in hepatitis or cirrhosis.^19^ Hence, the application of AFP for HCC diagnosis may be limited. Dual-phase computed tomography (CT) scan and magnetic resonance imaging only demonstrate a high diagnostic value in large nodules (>1-2 cm).^20^ Due to the unclear pathogenesis, the diagnosis and treatment of early-stage HCC are difficult, many patients are diagnosed at advanced stage. Hence, early HCC diagnosis is the basis for improving the survival rate of patients.^21^ Although tissue biopsy remains the gold standard for HCC diagnosis, its limited and invasiveness sampling are the main clinical obstacles. Therefore, it is of utmost importance to discover sensitive biomarkers for early HCC diagnosis.

Recently, exosome-derived circular RNAs, miRNAs, proteins, and so on can also be used as biomarkers for HCC. The results of quantitative polymerase chain reaction (q-PCR) analysis showed that circ-0051443 in the plasma- and tissue-derived exosomes of HCC patients was remarkably lower than that of control subjects, while a receiver operating characteristic curve (ROC) revealed that the patients with HCC could be distinguished from the controls by exosomal circ-0051443.^22^ Another ROC analysis indicated that the combination of AFP, miR-10b, -21, -122, and -200a could be considered as a superior strategy for early HCC diagnosis (area under curve [AUC] = 0.993).^23^ Liu et al^24^ found that the serum exosomal miR-125b level was markedly related to tumor growth, differentiation, and tumor node metastasis (TNM) stage. The decreased rates of overall survival (OS) and disease recurrence were observed in HCC patients with low exosomal miR-125b level, thus exosomal miRNA-125b may act as a prognostic biomarker for HCC patients. The high serum level of phosphoglycerate kinase 1 (PGK1) was closely associated with early recurrence and poor prognosis of HCC. Furthermore, the serum PGK1 level could complement AFP to enhance the specificity and sensitivity for predicting HCC recurrence.^25^ However, in this review, we emphasize exosomal lncRNAs.

Xu et al^17^ reported that the high ENSG00000258332.1 and LINC00635 levels in HCC were associated with OS, TNM stage, lymph node metastasis, and portal vein tumor emboli (all *P* < .05). Furthermore, the AUC of 2 lncRNAs combined with serum AFP was 0.894, indicating that the combination of serum exosomal AFP, LINC00635, and ENSG00000258332.1 may useful for the diagnosis and prognosis of HCC.^17^

Seventy-nine HCC patients were recruited in a prospective study.^26^ The data suggested that the higher levels of lncRNA-ATB and miR-21 were independent predictors of disease progression and mortality. Patients with higher circulating levels of exosomal lncRNA-ATB (≥0.0016) and miR-21 (≥0.09) had significantly lower OS and progression-free survival (*P* < .05). In sum, circulating exosomal lncRNA-ATB and miRNA-21 may serve as novel therapeutic targets and prognostic markers for HCC.

In another study, 35 chronic hepatitis C, 22 HCV-induced cirrhosis, and 10 HCV-related HCC patients were recruited in another study.^27^ In HCV-associated HCC patients, compared with chronic hepatitis C patients, the serum and exosomal expression levels of lncRNA-HEIH were elevated, but the ratio of lncRNA-HEIH expression was lower in serum than in exosomes, suggesting that lncRNA-HEIH may serve as a non-invasive serum biomarker for screening and diagnosing HCV-related HCC.

LINC00161 was noticeably upregulated in HCC patients’ serum specimens and exhibited excellent specificity and stability (fold change = 2.85, *P* < .001). Compared to controls, the expression levels of LINC00161 in the serum and exosomes were elevated in HCC patients (fold change = 4.27, *P* = .011). Their findings demonstrated that exosomal LINC00161 could serve as a promising biomarker for HCC.^28^

Lu et al^14^ showed that 3 lncRNAs, ENST00000457302.2, ENST00000440688.1, and ENSG00000248932.1, were upregulated in HCC patients compared to chronic hepatitis patients and control subjects. Furthermore, AFP combined with the 3 lncRNA panels showed an AUC of 0.870 for fingerprint functions in predicting HCC metastasis. Taken together, ENST00000457302.2, ENST00000440688.1, and ENSG00000248932.1 may be the promising biomarkers for distinguishing HCC from chronic hepatitis or healthy controls.^14^

Gramantieri et al^29^ identified that CASC9 and LUCAT1 knockdown enhanced invasion capability and cell motility in HCC cells and affected the epithelial-mesenchymal transition phenotypes. MiR-181d-5p could be directly sponged by LUCAT1. Both CASC9 and LUCAT1 were produced from the exosomes, and a high CASC9 level was correlated with tumor growth and postoperative HCC recurrence, implying its potential application as a non-invasive predictive biomarker for HCC recurrence.^29^

Huang et al^13^ reported the differential expression of 8572 lncRNAs and 9440 mRNAs in plasma exosomes between HCC patients and control subjects via RNA sequencing. Among them, a novel differentially expressed lncRNA, RP11-85G21.1, could promote HCC cell proliferation/migration by targeting and modulating miR-324-5p. In addition, the serum level of RP11-85G21.1 could distinguish AFP-negative HCC from liver cirrhosis and healthy controls with high accuracy.^13^

Cao et al^10^ showed that the expression of highly upregulated in liver cancer (HULC) in serum exosomes was associated with its level in HCC tissues, which was upregulated compared to healthy controls (n = 30). Upregulated expression of HULC induced invasion and proliferation, while suppressed apoptosis in HCC cells. Furthermore, q-PCR analysis revealed that HULC inhibited the expression of miR-372-3p, while Rab11a was determined to be a downstream target of miR-372-3p.^10^

Long noncoding RNA FAL1 could function as an oncogene in HCC and might be a predictive biomarker or a therapeutic target for HCC in the future. Long noncoding RNA FAL1 was overexpressed in the serum exosomes of HCC patients and could be transferred to HCC cells to enhance proliferation and migration abilities.^30^ As a competing endogenous RNA, lncRNA FAL1 could promote HCC growth and metastasis via competitive binding with miR-1236.

Hou et al^12^ identified AC012074.2, CTD-2116N20.1, LINC00501, RP11-136I14.5, and RP11-538D16.2 had prognostic values. Their expression levels were associated with OS and came from HCC-derived exosomes.^12^ According to the bioinformatics analysis and chromogenic in situ hybridization results, both RP11-538D16.2 and CTD-2116N20.1 worsen the prognosis of HCC patients by modulating the expression levels of proteins in exosomes.

It has been reported that the exosomal expression of RP11-583F2.2 in serum was upregulated in the HCC patients compared to HCV patients and control subjects.^15^ The findings demonstrated that miR-1298 and RP11-583F2.2 were more effective than AFP with regard to specificity and sensitivity. The selected miR-1298 and RP11-583F2.2 could decrease false negative errors compared with AFP, indicating that these biomarkers have diagnostic and therapeutic values in HCC patients.

Another research^16^ detected and compared SENP3-EIF4A1 expression in HCC patients and healthy controls. The findings demonstrated that SENP3-EIF4A1 was primarily encapsulated by exosomes and significantly decreased in HCC tissues and plasma exosomes from HCC patients (*P* < .05). Furthermore, exosomal SENP3-EIF4A1 was able to inhibit tumor growth *in vivo* and modulate ZFP36 expression via competitive binding with miR-9-5p.

In addition, RAB11A, miR-1262, and lncRNA-RP11-513I15.6 were capable of distinguishing HCC patients from chronic HCV patients and control subjects with excellent sensitivity and specificity.^11^ This study showed that by simultaneously measuring miR-1262, RP11-513I15.6, and AFP levels in serum exosomes, the diagnostic accuracy for early HCC detection was enhanced to 76.7% accuracy and 100% sensitivity.

Yao et al^31^ found that lnc-EPC1-4, lncZEB2-19, lnc-GPR89B-15, and lnc-FAM72D-3 were differentially expressed in HCC compared with hepatitis, cirrhosis patients, and healthy controls. Furthermore, the expression levels of lnc-EPC1-4 and lnc-GPR89B-15 were related to serum AFP levels. Lnc-FAM72D-3 knockdown promoted apoptosis and reduced cell viability, implying that it plays an oncogenic role in HCC. On the contrary, lnc-EPC1-4 overexpression induced cell apoptosis and suppressed cell proliferation, implying its tumor suppressive functions.

In conclusion, the expression level of exosomal lncRNA in HCC patients was different from that in control subjects or patients with other liver diseases. Thus, it may become a potential biomarker for the diagnostic and prognostic evaluation of HCC.

## Cholestatic Liver Injury

Chronic cholestatic liver diseases usually include primary sclerosing cholangitis (PSC) and primary biliary cholangitis (PBC). The pathogenesis of PSC remains unknown, and despite extensive research, liver transplantation remains the sole option apart from symptomatic treatment with ursodeoxycholic acid and immunosuppressants.^32,33^ Abnormal liver function test results and the presence of specific autoimmune markers are often the first indicators for PSC diagnosis. However, these are usually normal in PSC at the early stages. Cholangiography is regarded as gold standard method for the clinical diagnosis of this disease. Besides, PSC is a precancerous lesion, which may lead to the development of colon cancer, gallbladder neoplasia, and cholangiocarcinoma.^32^

To date, except for lncRNA, other RNA or protein can also serve as a biomarker. The EV proteomic signatures detected in the serum of PSC patients also showed promising values as a diagnostic tool.^34^ Stinton et al^35^ showed that proteinase-3-anti-neutrophil cytoplasmic antibodies performed better than atypical anti-neutrophil cytoplasmic antibodies for PSC diagnosis and avoided the challenges associated with indirect immunofluorescence assay testing.

Primary biliary cholangitis is a chronic progressive autoimmune disease that leads to the gradual destruction of intrahepatic bile ducts, fibrosis, cholestasis, and eventually cirrhosis if left untreated. Liver transplantation, the end-stage treatment for most patients resistance to ursodeoxycholic acid, offers good OS rates but moderate-to-high recurrence rates.^36^ Early biochemical markers include an increased serum alkaline phosphatase and γ-glutamyl transpeptidase, as well as a high bilirubin level at advanced stages.^37^ Serum anti-mitochondrial antibody (AMA) and PBC-specific antinuclear antibodies are recommended for patients with chronic (lasts >6) months intrahepatic cholestasis as the next diagnostic step.^37,38^ Anti-mitochondrial antibody is less suitable for acute hepatic injury,^39^ but it is highly specific and sensitive for PBC, especially chronic cholestasis. Although the diagnosis of PSC and PBC is systematic and effective, we still lack of specific biomarkers that can provide insight into the development of the disease.

The hepatic level of H19 was related to the severity of cholestatic injury in clinical PSC samples and fibrosis animal models. In addition, serum exosomal level of H19 was elevated during disease progression in liver cirrhosis patients and multidrug resistance 2 knockout (Mdr2^−/−^) mice. The H19-carrying exosomes in the primary cholangiocytes of wild-type mice, but not the exosomes in the cholangiocytes of H19^−/−^ mice, suppressed the hepatic expression of small heterodimer partners. More importantly, transplantation of H19-carrying serum exosomes from elderly Mdr2^−/−^ mice remarkably induce hepatic fibrosis in young Mdr2^−/−^ mice.^40^ Therefore, it can be inferred that exosomal H-19 plays an essential role in mediating cholestatic liver injury.

The progression of chronic cholestatic liver disease can be promoted by hepatic stellate cell (HSC) activation. Liu et al^41^ indicated that exosomal H19 in the cholangiocytes was associated with the progression of cholestatic liver fibrosis by inducing HSC activation and differentiation. H19 deficiency had an obvious protective effect on hepatic fibrosis in Mdr2^−/−^ and bile duct ligation (BDL) mice. In BDL-H19KO and DKO (Mdr2^−/−^H19^maternal Δ Exon1/+^) mice, transplantation of cholangiocyte-derived exosomal H19 was preferentially and rapidly taken up by HSC-derived fibroblasts and HSCs, thus promoting hepatic fibrosis. H19-enriched exosomes induced the proliferation of primary mouse HSCs and matrix formation in HSC-derived fibroblasts.

The activation of hepatic macrophages is a key driving force to promote cholestatic liver injury. Li et al^42^ reported that liver H19 levels were strongly associated with macrophage activation and liver fibrosis in BDL and Mdr2^−/−^ animal models and PBC or PSC patients. Cholangiocyte-derived exosomal H19 plays crucial roles in the activation, differentiation, and chemotaxis of macrophages via C-C chemokine receptor type 2/chemokine (C-C motif) ligand 2 signaling pathways.

Another research found that both serum and hepatic exosomal H19 levels were positively related to the severity of fibrotic liver damage in biliary atresia patients.^43^ Early diagnosis and Kasai hepato-portoenterostomy can help reduce the risk of cirrhosis and increase OS rates. However, due to the limited understanding of disease pathology and lack of reliable early diagnostic markers, biliary atresia represents the primary indicator for pediatric liver transplantation and the most common cause of advanced cirrhosis.^44^ H19 plays crucial roles in cholangiocyte growth and cholestatic liver injury in biliary atresia via regulation of let-7/HMGA2 and S1PR2/SphK2 axes. In short, the serum exosomal H19 may serve as a novel non-invasive therapeutic target and diagnostic biomarker for biliary atresia.^43^

## Viral Hepatitis

Chronic liver disease represents a major public health concern worldwide, which includes alcoholic liver diseases, fatty liver disease, chronic viral hepatitis. Viral hepatitis is an inflammation of the liver caused by different viruses. Although there are many treatment options, HBV and HCV infections remain major public health problems of the 21st century. Approximately 257 and 71 million people are living with HBV and HCV infections, respectively.^45^ Chronic HBV and HCV infections are the major risk factors for HCC.^46^

Assays for the measurement of HB-core antibody, HB-surface antibody, and HB-surface antigen in serum samples have been employed to detect patients with HBV exposure.^47^ Enzyme immunoassay is the initial test recommended worldwide for screening and diagnosing HCV, which determines anti-HCV antibodies in individuals. Histopathological assessment of a liver biopsy specimen is widely applied to evaluate the disease stage, which provides detailed information about liver scarring and inflammation. These conditions are the hallmarks of disease progression, which enables physicians to assess the aggressiveness of HCV infection.^48^

Only a portion of HCV-infected individuals may develop severe hepatic cirrhosis or HCC, and the outcome is difficult to be predicted. The expression level of serum lncRNA-HEIH was higher in HCV-associated HCC patients than in chronic hepatitis C patients.^27^ These findings may offer a new preventative method for predicting the risk of viral hepatitis progression.

## Alcoholic Liver Diseases

Alcoholic liver disease (ALD) is a common type of hepatic injury caused by heavy alcohol consumption. Excessive alcohol is a key contributor to >60 types of human diseases and is responsible for 3.3 million deaths annually, according to the Global Information System on Alcohol and Health.^49^ A major problem with the clinical diagnosis of ALD is that patients are often asymptomatic before developing serious and advanced diseases.^50^ The initial stage is a simple fatty liver, which ultimately develops into liver cirrhosis, liver fibrosis, and alcoholic hepatitis.

Lamichhane et al^51^ demonstrated that alcohol increased the vascularization activity of endothelial cell-derived EVs by upregulating pro-angiogenic lncRNA cargo (HOTAIR and MALAT1) and downregulating anti-angiogenic miRNA cargo (miR-106b). These findings could provide information on the role of exosomal lncRNA in alcohol-induced tumor progression.

## Discussion

In the present review, we have highlighted that exosomal lncRNA can serve as diagnostic, prognostic, predictive biomarkers for liver diseases (Table 1). Liquid biopsy for early diagnosis of liver diseases is an effective non-invasive strategy, since the occurrence and development of liver diseases are polygenic.

A good biomarker should possess the characteristics of stability, specificity, and sensitivity. The research on RNA as a biomarker mentioned above is only preliminary, which needs to be improved further. The specificity and sensitivity may be limited if only one circulating lncRNA is detected. Studies have shown that the combination of several exosomal lncRNAs or exosomal lncRNAs and traditional serum markers may greatly improve the diagnostic, prognostic, or predictive accuracy.

However, although exosomal lncRNAs are suggested to be biomarkers for several diseases, some issues need to be addressed before their clinical applications. First, the expression of lncRNAs is not only associated with the sample itself but is also affected by the internal reference gene and the extraction method used. Therefore, it is necessary to unify the standards among research institutions. Second, most of the studies are performed in small groups of patients. Hence, the correlation between certain exosomal lncRNAs and liver diseases with regard to diagnosis, prognosis, prediction specificity, and sensitivity needs to be validated in larger patient cohorts in the future. Third, the number of total lncRNAs in the exosome is low, which requires amplification before subsequent experiments.

In addition to the liver diseases mentioned above, drug-induced liver injury (DILI) has attracted significant attention worldwide in the past few decades, mainly due to its significant morbidity and mortality.^52^ However, only few studies have focused on exosomal lncRNA serving as biomarkers for DILI, which may be a promising field in the future.

In summary, although plenty of problems remain unsolved, exosomal lncRNA still plays a vital role in liver diseases. Thus, it is important to clarify the mechanisms of exosomal lncRNA and improve valid ways to extract or identify them, thus paving the way for clinicopathological examination.

## Figures and Tables

**Table 1.** The Application of Exosomal Long Noncoding RNAs in Diagnosis of Liver Diseases

lncRNA	Liver Disease	Source of Exosomes	Expression Levels	Application	Reference
ENSG00000258332.1 & LINC00635	HCC	Serum	Up	Diagnosis & prognosis	17
lncRNA-ATB	HCC	Serum	Up	Prognosis	26
lncRNA-HEIH	HCC	Serum	Up	Diagnosis & disease progression	27
LINC00161	HCC	Serum	Up	Diagnosis	28
ENSG00000248932.1 & ENST00000440688.1 & ENST00000457302.2	HCC	Plasma	Up	Predication the tumorigenesis and metastasis	14
CASC9 & LUCAT1	HCC	HCC cell	Up	Prognosis	29
RP11-85G21.1 (lnc85)	HCC	Plasma	Up	Diagnosis	13
lncRNA FAL1	HCC	Serum	Up	Diagnosis	30
CTD-2116N20.1 & RP11-538D16.2	HCC	HCC tissue		Prognosis	12
lncRNA-RP11-583F2.2	HCC	Serum	Up	Diagnosis	15
SENP3-EIF4A1	HCC	Plasma & HCC tissue	Down	Diagnosis	16
lncRNA-RP11‐513I15.6	HCC	Serum	Up	Diagnosis & prognosis	11
lnc-EPC1-4 Lnc-FAM72D-3	HCC	Serum	Down up	Diagnosis & prognosis	31
H19	PSC	Serum	Up	Disease progression	40
H19	BA	Serum	Up	Diagnosis	43

HCC, hepatocellular carcinoma; lncRNA, long noncoding RNA; PSC, primary sclerosing cholangitis.
